# Gait speed and its associated factors among older black adults in Sub-Saharan Africa: Evidence from the WHO study on Global AGEing in older adults (SAGE)

**DOI:** 10.1371/journal.pone.0295520

**Published:** 2024-04-18

**Authors:** Phyllis Tawiah, Paulina Boadiwaa Mensah, Solomon Gyabaah, Atinuke Olusola Adebanji, Emmanuel Konadu, Isaac Amoah

**Affiliations:** 1 Department of Medicine, School of Medicine and Dentistry, College of Health Sciences, Kwame Nkrumah University of Science and Technology, Kumasi, Ghana; 2 University Hospital, Kwame Nkrumah University of Science and Technology, Kumasi, Ghana; 3 Komfo Anokye Teaching Hospital, Kumasi, Ghana; 4 Department of Statistics and Actuarial Science, Kwame Nkrumah University of Science and Technology, Kumasi, Ghana; 5 School of Public Health, Kwame Nkrumah University of Science and Technology, Kumasi, Ghana; 6 Department of Biochemistry and Biotechnology, Kwame Nkrumah University of Science and Technology, Kumasi, Ghana; Instituto Nacional de Geriatria, MEXICO

## Abstract

Gait speed is an essential predictor of functional and cognitive decline in older adults. The study aimed to investigate the gait speed of older adults in Ghana and South Africa and to determine its associated factors, as the Sub-Saharan representatives in the World Health Organization’s Study on Global AGEing in Older Adults (SAGE). A secondary analysis of data from the SAGE study which consists of nationally representative data involving participants aged ≥50+ years with smaller samples of younger adults aged 18–49 years in Ghana and South Africa was conducted. SAGE study employed a multistage, stratified clustered sample design and involved the use of a standardised questionnaire to obtain participants’ (n = 5808) demographic, anthropometric and gait speed information. The standard 4 metre-gait speed was used. Median gait speed for the study group, which comprised African/Black participants aged ≥50+ years was 0.769(Q1 = 0.571, Q3 = 0.952)m/s for males and 0.667 (Q1 = 0.500,Q3 = 0.833)m/s for females. For every unit increase in age, the odds of being in a higher-ranked gait speed category was 0.96(95%CI 0·96, 0·97, *p<0*.*001*) times that of the previous age. Females had odds of 0.55 (95%CI 0.50, 0.61, *p<0*.*001*) of recording higher gait speed, as compared to males. Rural dwellers had odds of 1.43 (95%CI 1.29, 1.58, *p < 0*.*001*) of being in a higher-ranked category of gait speed compared to urban dwellers. Underweight (OR = 0.85, 95%C1 = 0.73–1.00, *p<0*.*05*) and obesity (OR = 0.53, 95%CI = 0.46–0.61, *p<0*.*001*) were associated with slower gait speed. Amongst functional indices, the World Health Organization Disability Assessment Schedule (WHODAS) score was the biggest determinant of gait speed. Having a “Severe/Extreme” WHODAS score had the strongest association with gait speed (OR = 0.18, 95%CI = 0.14–0.23, *p<0*.*001*). These gait speed results provide an essential reference for older adults’ care in Ghana and South Africa.

## Introduction

Globally, countries are observing an increased shift in population demographics characterised by a surge in ageing population [1,2]. For example, in 2020, older adults aged 60 years and above outnumbered younger children in the ≤5 years old group [1]. The proportion of older adults aged 60 years and above was estimated to be around 1 billion in 2020 and this number is projected to increase to 2.1 billion by 2050 worldwide [1]. Regarding geographical stratification, Sub-Saharan Africa is expected to record the second fastest increase, with older adults aged 65 years and over expected to grow from 32 million in 2019 to 101 million in 2050 [2]. Additionally, it has been projected that 80% of older adults aged 60 years and above will be living in low and middle-income countries including Sub-Saharan Africa [1].

Factors including advances in medical care treatment, personal hygiene, public health and nutritional interventions have been reported as the underpinning reasons driving the apparent increase in the global ageing population [3]. That notwithstanding, life expectancy in developing countries, notably Sub-Saharan Africa, lags behind that of developed countries [4,5]. For example, the life expectancy for Ghana and South Africa stand at 64 and 65 years respectively whereas that of USA, United Kingdom, Australia and New Zealand stand at 77, 81, 83 and 82 years respectively [5]. Ageing presents its attendant physiological and functional changes including reduced absorption of key nutrients [6], loss of muscle mass and degenerative changes in the joints [7]. Consequent to these changes, it has been well established that the gait speed of older adults is affected by ageing [7,8].

Gait speed is an emerging clinical marker that transcends disease and functional status. Slower gait speed is a reliable predictor of functional decline [8], risk of falling [9], hospitalisation likelihood [10,11] and survival among community-dwelling adults [11]. Thus, it has gained a reputation as a vital sign in older adults for assessing their functional status. Interventions including balance and resistance training have demonstrated improvement in gait speed across all age groups in older adults [12].

A recent systematic review of published literature assessed gait speed measurement in older adults and its potential suitable adoption for use in clinical setting [13]. The authors reported that even though most of the studies employed walking distances of 2.4 and 15m, the commonly reported gait speed for the participants was 4m [13]. Gait speed is easily performed and can be incorporated into routine geriatric clinics. It has been studied extensively in the last two decades [14] with a consistent ability to predict significant outcomes such as life expectancy in developed countries. As a result of the limited proportions of black populations in these studies and the unequal socioeconomic environment, the results cannot be extrapolated to blacks living in low-income Sub-Saharan Africa. Consequently, it is safe to conclude that the impact of gait speed and the associated factors on ageing outcomes have not been well studied in Sub-Saharan Africa.

Numerous studies have established normative data for normal gait speed and associated factors. Factors that have been identified to impact gait speed adversely include being of female gender [15], older age [14], presence of existing comorbidity particularly cardiovascular disease [16], poor socioeconomic status [16], increased sedentary lifestyle [16], higher level of illiteracy and having difficulty in one or more instrumental activities of daily living [16]. Unfortunately, these studies are mostly conducted in developed countries and predominately non-black populations. There is, therefore, a paucity of nationwide data on gait speed for community-dwelling older adults in the Sub-Saharan region. The World Health Organization’s Study on Global AGEing in Older Adults (SAGE) is a nationally represented longitudinal study in six low- and middle-income countries to provide reliable data on ageing. The countries studied include China, Ghana, India, Mexico, the Russian Federation, and South Africa. We therefore aimed to determine the normal gait speed for community-dwelling older adults in Ghana and South Africa who represented Sub-Saharan Africa and to assess factors that impact gait speed in this population. This data will guide the clinical care of older adults and provide factors that, when modified, could limit frailty and mortality in the long run, in this subregion.

## Methods

### Study design

This was a cross-sectional study using data obtained from the World Health Organization (WHO) survey on Global Ageing and Adult Health (SAGE) Wave 1 for Ghana and South Africa. The SAGE baseline data, Wave 0, was collected from 2002 to 2004, as part of WHO’s World Health Survey [17]. The SAGE Wave 1 used participants recruited from the Wave 1 and additional participants between 2007 and 2010 [17], collecting a comprehensive dataset on the health and well-being of adults in six low and middle-income countries: China, Ghana, India, Mexico, Russian Federation and South Africa.

### Sampling technique

The WHO SAGE used a multistage-clustered sampling technique, which comprised of a two-stage probability that generated national and sub-national estimates with adequate accuracy. The sample was stratified by provinces, locality type (urban and rural) and population group (including Black, Coloured, Indian or Asian, and White) for South Africa [18] and by region and type of locality for the Ghanaian study [19].

In Ghana, the sample was stratified by administrative region (Ashanti, Brong Ahafo, Central, Eastern, Greater Accra, Northern, Upper East, Upper West, Volta and Western) and type of locality (urban/rural) resulting in 20 strata, which is nationally representative.

The Census Enumerated Areas (CEA) of the 2010 Population and Housing Census was used as the sampling frame [20]. The number of Enumeration Area (EA) to be selected from each strata was based on proportional allocation (determined by the number of EA in each strata specified on the census frame). EAs were then selected from each stratum with probability proportional to size; the measure of size being the number of individuals aged 50 years or more in the EA [20].

In South Africa, strata were defined by the nine provinces: (Eastern Cape, Free State, Gauteng, Kwa-Zulu Natal, Limpopo, Mpumalanga, North West, Northern Cape and Western Cape), locality (urban or rural), and predominant race group (African/Black, White, Coloured and Indian/Asian), as not all combinations of stratification variables were possible. The number of EAs to be selected from each strata was based on proportional allocation (determined by the number of EAs in each strata-specified on the Master Sample) [21]. EAs were then selected from each strata with probability proportional to size; the measure of size being the number of individuals aged 50 years or more [21].

### Sample size

**Fig 1:**
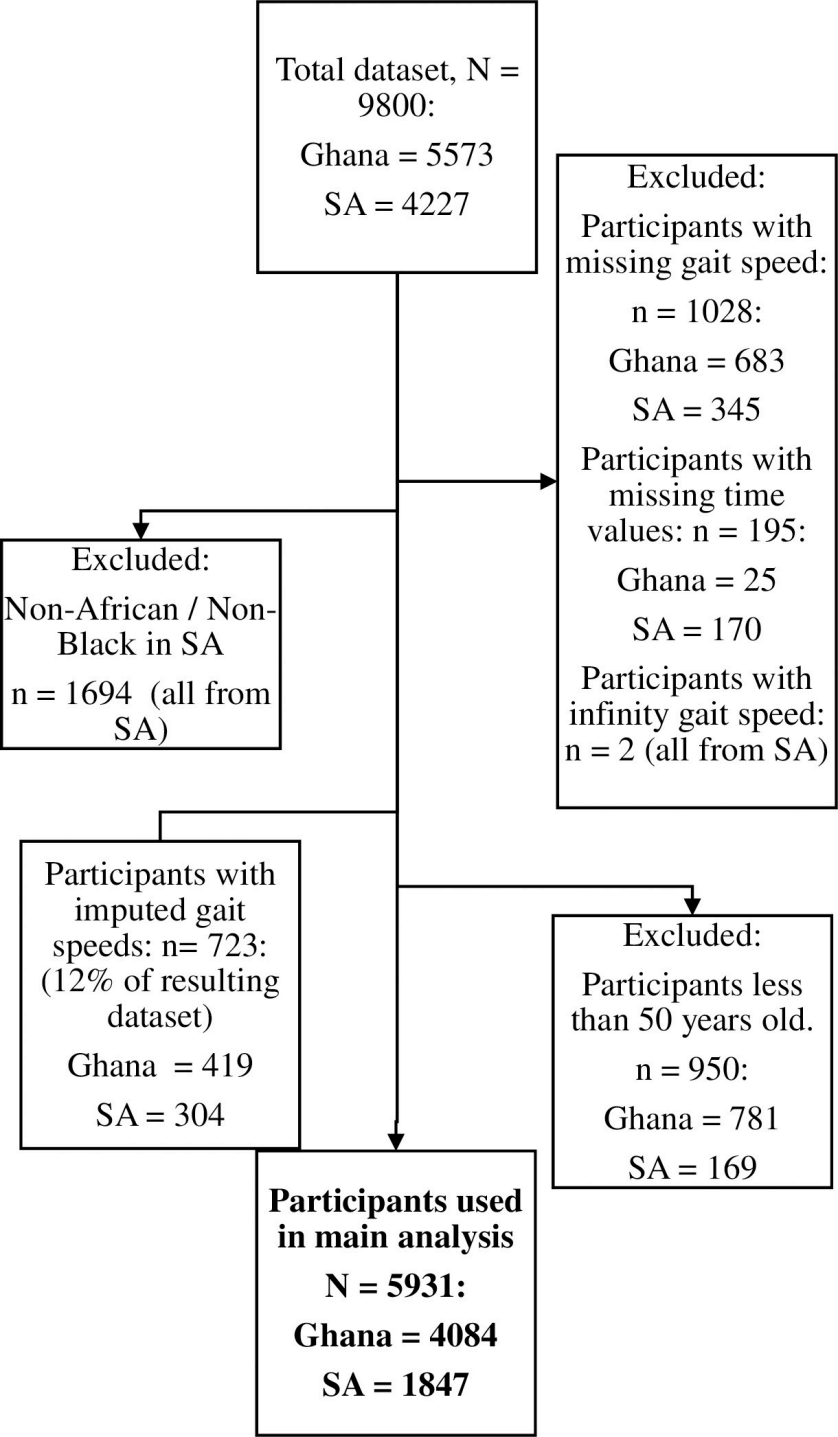
Flow diagram showing the numbers of participants recruited for the study. The 723 participants with imputed gait speeds were originally outliers (had gait speeds either below the 5^th^ percentile or above the 95^th^ percentile).

An initial total of 9800 entries were collected, with 5573 from Ghana and 4227 from South Africa. A total of 1028 entries had “Not Applicable”/“Cannot walk” for gait speed, which comprised 683 from Ghana and 345 from South Africa. These 1028 entries were analyzed separately in the Suppoprting information (S1 Table). Two entries from South Africa had exactly zeros recorded for the time taken to cover the distance of 4m. This gave them a gait speed of infinity, as this is unrealistic, these two entries were also dropped from the data. Also, 195 participants had missing values for the time, and these entries were dropped. The data was filtered to exclude participants less than 50 years old as the study focused on older adults. Additionally, 1694 non-black participants from South Africa were excluded. The rationale for excluding non-black participants from South Africa is explained in the Supporting information (S2 Table). The 5^th^ and 95^th^ percentile value for gait speed were found for both countries respectively as (0.36 and 1.24m/s for Ghana) and (0.37 and 1.33m/s for South Africa). Entries below the 5^th^ percentile and above the 95^th^ percentile for each country were imputed with the respective values. The final sample size composed of 5931 entries: 4084 from Ghana and 1847 from South Africa (Fig 1).

### Study area

Ghana’s total population per the 2010 population and housing census was 24,658,823 with a sex ratio of 95.2 males per 100 females [22]. Quite a huge number (38.3%) of Ghana’s population is less than 15 years, 49.5% is 15–49 years, and; 12.2% is aged 50 years and above [22]. The proportion of adults 60 years and older were 6.7% and 65 and above year-olds were 4.7%. More than half of this population is living in urban areas (Ghana Statistical Service, 2010). Agriculture is the most predominant occupation found in Ghana with most people engaged in small-scale farming. Specifically, 41.2% of the economically active population is skilled agricultural, forestry and fishery workers; about 21% is also engaged as service and sales workers, while 15.2% are craft and related trade workers [23]. Ghana is a lower-middle-income country with an average per capita income of GH¢5,347(about US$1,353) [24]. Health spending as a percentage of GDP is 5.4% in 2013, higher than the WHO recommendation of 5% [25].

South Africa’s total population per the 2011 Population and Housing Census stood at 51,770,560 with a sex ratio of 95 per 100 females [26]. Functional age group patterns show that the 0–14 age group for both males and females decreased whilst those of the economically active population (15–64) increased over time. Overall, 4.1% of the total male population in South Africa was above 64 years and 6.5% of the total female population was above 64 years [26]. South African population consist of different race: 79.2% black, 8.9% coloured, 2.5% Indian/Asian and 8.9% white [26]. South Africa is a middle-income country with an average GDP per capita of US$7441.2 in 2013 [27].

### Measures

#### Demographic characteristics

Demographic data were collected using face-to-face interviews with structured questionnaires, on age, sex, marital status, level of education, residence (rural/urban) and income.

#### Anthropometry

Height and weight were measured with the use of a stadiometer and a routinely calibrated electronic weighing scale respectively. Body mass index (BMI) was calculated as weight in kilograms divided by height in meters squared. BMI was categorized as the following:<18.5 kg/m^2^ (underweight), 18.5–24.9 kg/m^2^ (normal weight), 25.0–29.9 kg/m^2^ (overweight), > 30 kg/m^2^ obesity [28].

#### Gait speed

A distance of 4 metres was measured and marked and participants were asked to walk that distance. The gait speed was then calculated as 4 metres (m) divided by the time taken by the respondent to walk that distance. The 4-m gait speed test is a simple, easy to perform and reliable functional performance measure [29].

#### Disability

Disability was assessed using the composite World Health Organization Disability Assessment Schedule 2.0, inverted (WHODASi) disability questionnaire. It captures 6 domains of day-to-day functioning in the last 30 days. It includes functional status questions for activities of daily living and instrumental activities of daily living. For this study, the WHODAS-related variables are a series of questions that start with the following preamble: “Overall in the last 30 days, how much difficulty…”, followed by various parameters/tasks. The respondents’ answers were re-coded with 0 equivalent to “none”, 1 equivalent to “mild”, 2 to “moderate”, 3 to “severe” and 4 to “extreme”. Across the 12 variables, a respondent could score a minimum of 0 and a maximum of 48.

#### Comorbidities

The comorbidities examined in this study were angina, diabetes mellitus and hypertension. Respondents who reported a prior diagnosis of angina or angina pectoris or who had experienced any pain or discomfort in the chest when walking were regarded as having angina. Blood pressure was measured 3 times in SAGE surveys with less than one-minute intervals using standard protocols. Mean systolic and diastolic pressure were subsequently obtained. Hypertension was a dichotomous variable and was defined as at least either one of the following: mean systolic blood pressure >140 mmHg, diastolic blood pressure >90 mmHg, and self-reported diagnosis of hypertension [28]. The presence of diabetes mellitus was based on self-report as to whether or not the participant was ever diagnosed with the condition.

### Ethical approval

Ethical approval for the SAGE study was obtained from the World Health Organization, Geneva, Switzerland; the Ethics Committee and the local Ethics Review Committee for each country involved. In Ghana and South Africa, ethical approval was sought from the University of Ghana Medical School, Accra, Ghana; Institutional Review Board and the Human Sciences Research Council, Pretoria, South Africa, respectively. This approval covered all procedures undertaken as part of the study. In addition, written informed consent was freely obtained from each participant. SAGE country teams maintain confidential records of participants’ consent. The authors do not have access to information that could identify individual participants involved in the study.

### Statistical analysis

Data were analysed using R Version 4.1.2. Data normality was assessed with the Kolmogorov-Smirnov normality test which returned p< 0.001, indicating non-normality. Consequently, the median was used as the measure of central tendency for the descriptive statistics. The gait speeds were grouped and ordered as “below 25th percentile”, “between 25th and 75th percentile”, and “above 75th percentile” from the lowest-ranked group to the highest-ranked group, based on the values obtained at the 25th and 75th percentiles of the recorded gait speeds. A multivariable ordinal logistic regression model was used to determine the predictors for gait speed. Variables were selected via a stepwise variable selection method involving a hybrid of both forward and backward selection methods and based on Akaike information criterion (AIC). Collinearity was assessed using the variance inflation factor (VIF). The threshold of statistical significance was p <0.05.

## Results

### Overview of demographics and socioeconomic indices for the study group (African/Black population, aged 50 years and above, in Ghana and South Africa combined)

A total of 5931 participants were studied. In terms of age stratification, 40% and 46% of the participants were between the ages of 50–59 years in Ghana and South Africa respectively, and about 50% of them were in the 60–79 age groups for both countries. There was a higher proportion of male participants than female participants (52% male and 48% female respectively) from Ghana with the opposite (39% male and 61% female) observed for South Africa. The majority (59%) of the participants in Ghana resided in rural areas, whereas, for South Africa, the opposite (44%) was noted. Most participants were “cohabiting/currently married” in both countries. Income was almost equally distributed across the five quintiles in Ghana, but South Africa had the highest proportion of participants in the 1^st^ income quintile (Table 1).

**Table 1:** Overview of socio-demographic characteristics of African/Black population in Ghana (n = 4084) and South Africa (n = 1847).

Characteristic	Ghana N (%)	South Africa Black N (%)
**Age groups (years)**		
50 to 59	1 639 (40%)	845 (46%)
60 to 69	1 149 (28%)	579 (31%)
70 to 79	917 (22%)	299 (16%)
80 and above	379 (9·3%)	124 (6·7%)
**Gender**		
Male	2 140 (52%)	719 (39%)
Female	1 944 (48%)	1 128 (61%)
**Years of education**		
0	2 264 (56%)	695 (38%)
1 to 12	1 435 (36%)	1 031 (56%)
12 and above	327 (8·1%)	120 (6·5%)
Unknown	58 (0.01%)	1 (0.0005%)
**Residence**		
Urban	1 662 (41%)	1 024 (56%)
Rural	2 422 (59%)	821 (44%)
Unknown	0 (0%)	2 (0.001%)
**Marital status**		
cohabiting/currently married	2 328 (57%)	865 (47%)
single/separated	623 (15%)	409 (22%)
widowed	1 112 (27%)	537 (29%)
don’t know	21 (0·5%)	36 (1.9%)
**Income (in quintiles)**		
1st	812 (20%)	482 (26%)
2nd	804 (20%)	453 (25%)
3rd	802 (20%)	382 (21%)
4th	837 (21%)	331 (18%)
5th	824 (20%)	189 (10%)
Unknown	5 (0.001%)	10 (0.005%)

The median gait speed by age groups and gender were generally similar for both the South African and Ghanaian participants (Table 2).

**Table 2:** Median gait speed by age groups and gender.

Gender	Age	Ghana/Median gait speed(Q1,Q3) (m/s)	South Africa/ Median gait speed(Q1,Q3) (m/s)	Combined/ Median gait speed(Q1,Q3) (m/s)
**Male**	**50–59**	0.800 (0.645,1.000)	0.800 (0.588,1.000)	0.800 (0.635, 1.000)
	**60–69**	0.769 (0.57,0.952)	0.755 (0.521, 1.000)	0.769 (0.563, 0.976)
	**70–79**	0.690 (0.548,0.870)	0.727 (0.563,0.994)	0.702 (0.548, 0.889)
	**80+**	0.656 (0.500, 0.842)	0.615 (0.435,0.741)	0.656 (0.488, 0.816)
**Female**	**50–59**	0.741 (0.571,0.909)	0.667 (0.500,0.889)	0.696 (0.548, 0.909)
	**60–69**	0.667 (0.506,0.851)	0.667 (0.500,0.833)	0.667 (0.500,0.851)
	**70–79**	0.580 (0.444,0.784)	0.667 (0.444,0.889)	0597 (0.444,0.800)
	**80+**	0.479 (0.376,0.667)	0.556 (0.423,0.769)	0.500 (0.398,0.690)

### Socio-demographic characteristics, comorbidities and functional indices amongst study population

In the Ghana population, 40% of the participants were aged 50–59 years whereas 46% of their South African counterparts were in that age category (Table 3). For those aged 60 to 79 years, Ghana had 50% whereas South Africa recorded 47%. Majority of the participants from Ghana were males (52%) whereas females (61%) were the predominant participants in the South African study population.

**Table 3:** Overview of socio-demographic, anthropometric, comorbidities and functional indices amongst study population.

	Main Study Population n (%)	
Socio-demographic and Anthropometric Indices	Ghana (N = 4084)	South Africa (N = 1847)
**Age groups (years)**		
50 to 59	1 639 (40%)	845 (46%)
60 to 69	1 149 (28%)	579 (31%)
70 to 79	917 (22%)	299 (16%)
80 and above	379 (9·3%)	124 (6·7%)
**Sex**		
Male	2 140 (52%)	719 (39%)
Female	1 944 (48%)	1 128 (61%)
**Residence**		
Urban	1 662 (41%)	1 024 (56%)
Rural	2 422 (59%)	821 (44%)
Unknown	-	2 (0.1%)
**BMI**		
Underweight	628 (15%)	128 (6·9%)
Normal Weight	2 282 (56%)	485 (26%)
Overweight	773 (19%)	481 (26%)
Obese	401 (9·8%)	753 (41%)
Unknown	-	-
**History of Disease**	**Ghana (N = 4084)**	**South Africa (N = 1847)**
**History of Diabetes Mellitus**		
no	3 930 (96%)	1 689 (93%)
yes	152 (3·7%)	129 (7·1%)
Unknown	2	29 (1.6%)
** History of Hypertension**		
no	3 599 (88%)	1 403 (76%)
yes	482 (12%)	440 (24%)
Unknown	3	4 (0.2%)
**History of Angina**		
no	3 164 (78%)	1 565 (85%)
yes	918 (22%)	281 (15%)
Unknown	2	1 (0%)
** Functional Index**	**Ghana (N = 3 935)**	**South Africa (N = 1626)**
**WHODAS score**		
none or mild	1 250 (32%)	656 (40%)
moderate	2 183 (55%)	811 (50%)
severe or extreme	502 (13%)	159 (9·8%)

Differences in N are due to filtering out missing entries.

Most (56%) of the Ghanaian participants has a normal BMI whereas 26% of the South African participants were in the normal BMI group. Most (67%) of the South African participants had higher BMI (overweight and obese) whereas 28.7% of the Ghanaian population were in that BMI category.

In Ghana, 13% had poorer WHODAS scores (“severe or extreme”) as compared to 9.8% recorded for the South Africa population. Higher percentages of positive histories of diabetes mellitus and hypertension were recorded for the South Africa group compared to the Ghana group. Higher percentages of positive histories of angina was recorded in the Ghana group compared to the South Africa group (Table 3).

### Association between selected variables and gait speed

The median gait speed was 0.769 (Q1 = 0.571, Q3 = 0.964) m/s for males and 0.661 (Q1 = 0.500, Q3 = 0.833) m/s for females. Amongst the demographic variables analyzed, Age had the strongest association with gait speed, followed by Gender and Residence in that order (Table 4).

**Table 4:** Association between selected variables and gait speed.

Unadjusted Model				Adjusted Model	
Characteristic	Median gait speed (Q1,Q3) (m/s)	OR (95% CI)	p-value	OR (95% CI)	p-value
**Age**		0.96 (0.96,0.97)	p<0.001	0·97 (0·96,0·98)	p<0·001
**Gender**			p<0.001		
Male	0.769 (0.571,0.964)	*1*		*1*	
Female	0.661 (0.500,0.833)	0.53 (0.48,0.58)		0.64 (0.58,0.72)	p<0·001
**Residence**			p<0.001		
Urban	0.667 (0.500,0.870)	*1*			
Rural	0.727 (0.556,0.930)	1.38 (1.25,1.52)		1.34 (1.20,1.50)	p<0·001
**Height**	0.667 (0.500,0.870)	1.04 (0.95,1.15)	p = 0.38	1.00 (0.90,1.11)	p = 0.99
**BMI**			p<0.001		p<0.001
Normal Weight	0.741(0.563,0.930)	*1*		*1*	
Underweight	0.667 (0.513,0.889)	0.77 (0·57,1·04)		0·92 (0.78,1·08)	
Overweight	0.727 (0.556,0.930)	0.99 (0·82,1·21)		0·99 (0·86,1·13)	
Obese	0.606 (0.471,0.816)	0.53 (0·46,0·61)		0·55 (0·47,0·65)	
**Income (in quintiles)**			p<0.005		p = 0.24
1st	0.727 (0.556,0.930)	*1*		*1*	
2nd	0.702 (0.533,0.930)	0.87(0.75,1.00)		0.92 (0.79,1.08)	
3rd	0.667 (0.500,0.889)	0·75 (0.64,0.87)		0.83 (0.71,0.98)	
4th	0.690 (0.532,0.889)	0·79 (0.68,0.91)		0.89 (0.75,1.05)	
5th	0.690 (0.519,0.909)	0·82 (0.70,0.95)		0.93 (0.77,1.11)	
**Country**			p = 0.47		p<0.005
Ghana	0.702 (0.548,0.909)	*1*		*1*	
South Africa	0.667 (0.500,0.952)	0.96 (0.87,1.07)		1.23 (1.08,1.41)	
**WHODAS**					
None or Mild	0.769 (0.580,0.952)	*1*		*1*	p<0.001
Moderate	0.597 (0.455,0.816)	0.39 (0.35,0.44)		0.57 (0.51,0.64)	
Severe or Extreme	0.500 (0.398,0.681)	0.21 (0.16,0.26)		0.28 (0.24,0.34)	
**History of Hypertension**					
Yes	0.667 (0.500,0.833)	*1*		*1*	
No	0.727 (0.556,0.952)	1.24 (1.08,1.41)	p = 0.002	0.99 (0.81,1·20)	p = 0.89
**History of Diabetes Mellitus**					
Yes	0.656 (0.465,0.800)	*1*		*1*	
No	0.690 (0.541,0.909)	1.73 (1.38,2.18)	p<0.001	1·20 (0.93, 1·54)	p = 0·57
**History of Angina**					
Yes	0.667 (0.500,0.851)	*1*		*1*	
No	0.702 (0.541,0.930)	1.33 (1.18,1.50)	p<0.001	1·29 (1·08, 1·54)	p<0.005

Q1 = Lower quartile, Q3 = Upper Quartile, OR = Odds Ratio; CI = Confidence Interval; *1* = Reference; p-value < .05 was deemed significant. Global p-values reported for variables with more than two levels.

Adjusting for all the other variables, for every unit increase in age, the odds of being in a higher-ranked gait speed category was 0.97 (95%CI 0·97, 0·97, *p<0*.*001*) times that of the previous age. Females had odds of 0.64 (95%CI 0.58, 0.72, *p<0*.*001*) of being in a higher-ranked category of gait speed, as compared to males. Rural dwellers had odds of 1.34 (95%CI 1.20, 1.50, *p<0*.*001*) of being in a higher-ranked category of gait speed as compared to urban dwellers. Income did not have a statistically significant association with gait speed.

Amongst the clinical and functional indices, both BMI and WHODAS scores have strong associations with gait speed (*p<0*.*001*). Participants in a higher WHODAS score category had lower odds of being in a higher gait speed category. Being underweight, overweight or obese were all associated with lower odds of being in a higher gait speed category. Obese participants (OR = 0.55, 95%CI = 0.47–0.65) had the lowest odds of being in a higher gait speed category as compared to the participants in the other BMI categories. After adjusting for all the other variables, only a positive history of angina was associated with lower odds of being in a higher gait speed category, amongst the three diseases analyzed. Height did not have a statistically significant association with gait speed.

The country of the participant was not statistically significant as a predictor of gait speed category in the unadjusted model, but was significant after adjusting for the other selected variables (Table 4).

### Association between gait speed and relevant socio-demographic variables of older black adults in Sub-Saharan Africa

**Fig 2:**
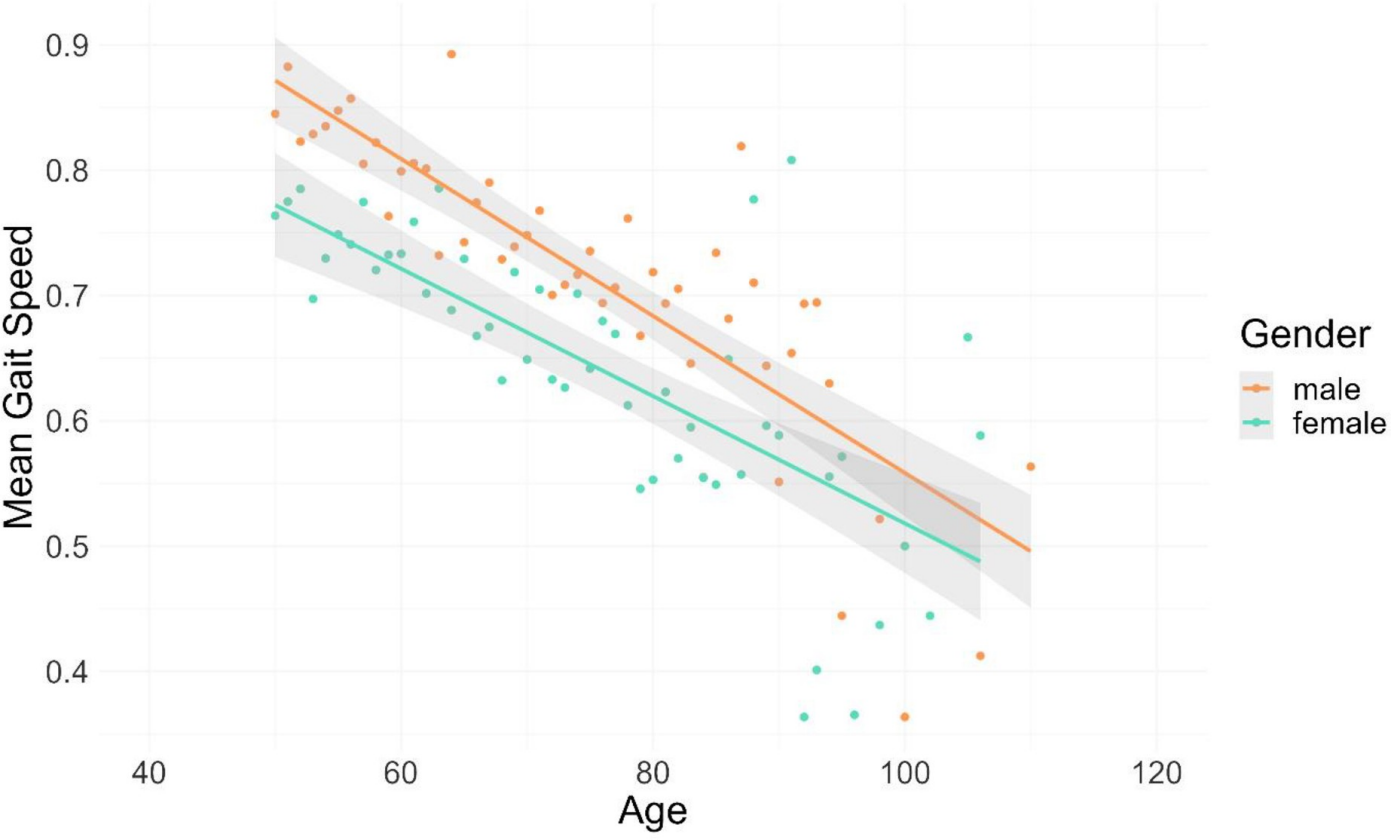
Age versus mean gait speed grouped according to the sex of respondents. The straight lines represent linear regression models and the shaded gray areas represent the confidence intervals.

The relevant socio-demographic variables were found to be Age, Gender and Residence. A slower gait speed with increasing age was noted in this study (Fig 2). Men had higher gait speeds compared to women even with advancing age (Fig 2).

**Fig 3:**
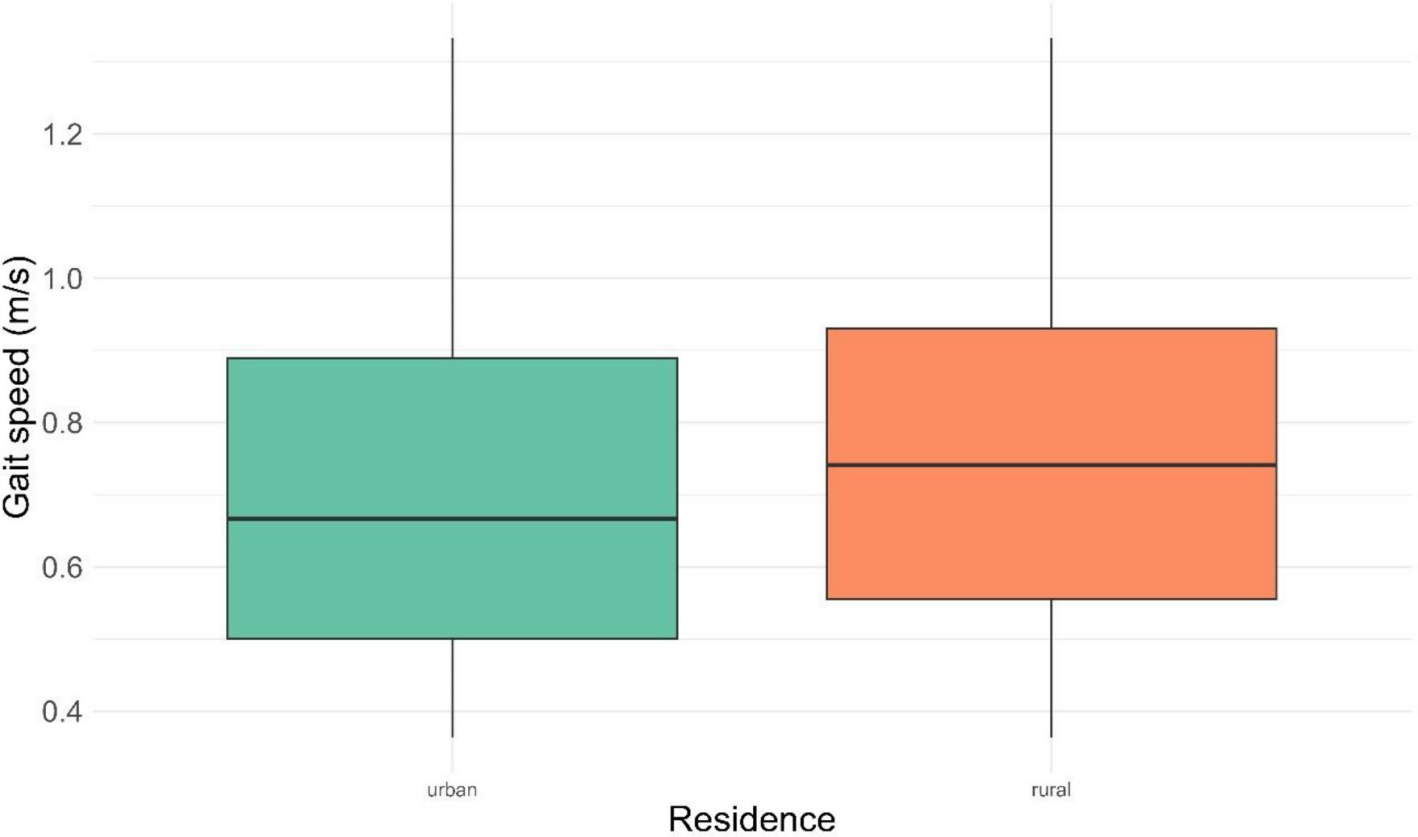
Residence of respondents versus gait speed. The middle bar of the boxplot represents the median. The lower and upper borders of the box represent the 25th and 75th percentiles respectively. The upper and lower ends of the whiskers represent maximum and minimum values respectively.

Rural dwellers had higher gait speeds than urban dwellers (Fig 3).

### Association between gait speed and functional and anthropometric indices of older Black adults in Sub-Saharan Africa

**Fig 4:**
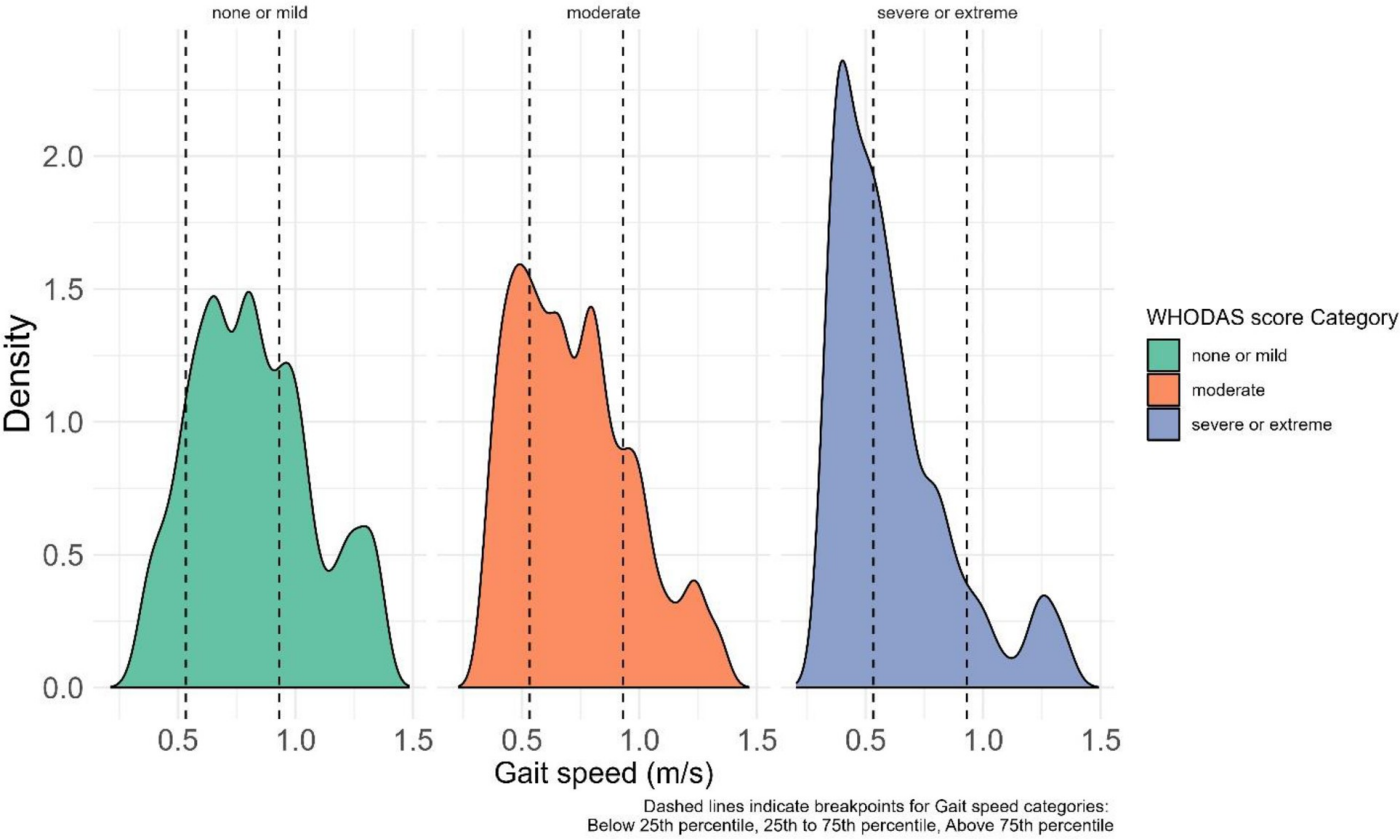
WHODAS score category versus gait speed. The ridges represent the WHODAS score categories. The height and peaks of each ridge is equivalent to the density of participants in that category. The dashed lines show the cut-off points for the various gait speed categories.

Respondents in lower WHODAS score categories had higher gait speeds than those with higher WHODAS scores (Fig 4).

**Fig 5:**
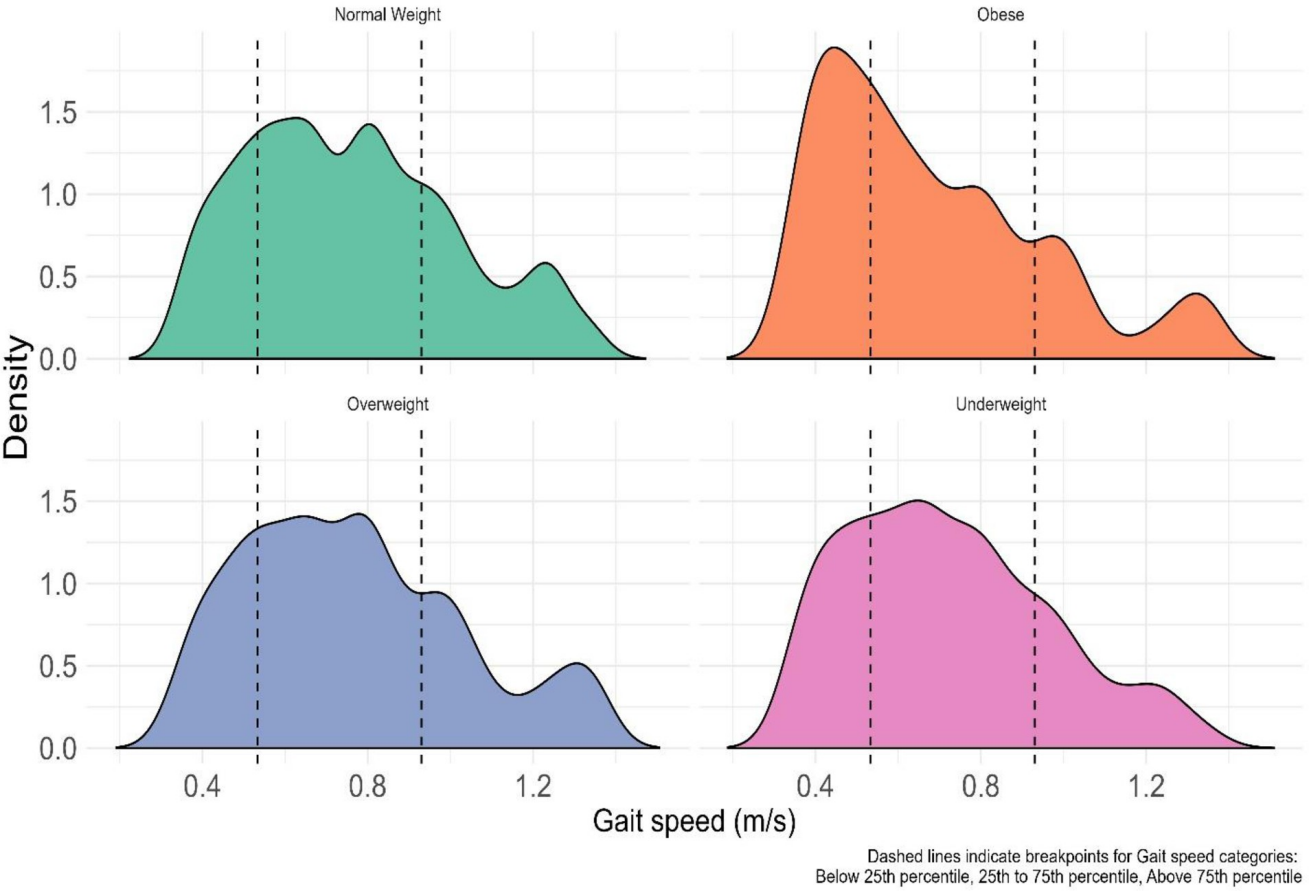
BMI Category versus gait speed. The ridges represent the WHODAS score categories. The height and peaks of each ridge is equivalent to the density of participants in that category. The dashed lines show the cut-off points for the various gait speed categories. Normal Weight—BMI: 18.5–24.9, Underweight—BMI less than 18.5, Overweight—BMI: 25.0–29.9, Obese—BMI: 30 and above.

In addition, respondents with normal weight had higher gait speeds than those underweight, overweight or obese. Respondents who were obese had the lowest gait speeds across the age groups, followed by those who were underweight (Fig 5).

## Discussion

In this study, we identified the main factors associated with a lower category of gait speed amongst older Ghanaians and black South Africans as increasing age, being of female gender, living in an urban area, having a high WHODAS score, being obese, and having a positive history of angina. This is the first of its kind in the Sub-Saharan region. Even though the findings of this study confirm what others have done in other parts of the world, the uniqueness of this work includes that a nationally-represented data on black population from SSA was used. In Ghana, there is currently a paucity of data on gait speed in older adults. The WHO SAGE data is the only nationally-represented data currently available, thus, creating the opportunity for its assessment to provide a normative data for both clinical and research reference. This will provide solid reference for people looking for information on black populations/SSA which is different for other races including for example those in the USA.

In this present study, descriptive analysis of the 1028 respondents (representing 10.5% of the total dataset of 9800 entries) showed higher percentages of comorbidities especially histories of diabetes mellitus, angina and hypertension, and higher severity in disability, based on WHODAS scores, in these respondents as compared to the main analysis population as shown in the Supporting information (S1 Table). These comorbidities and their associated complications are clinically notable for gait impairment. It is therefore not surprising that respondents with these comorbidities would not be able to walk or would refuse to walk. This confirms earlier findings by Cesari et al. who reported that, older community dwelling Italian adults living in the Sirente geographic area with significant comorbidities reported lower gait speed compared to their counterparts without any comorbidities [30].

The median gait speed ranged from 0.800 (0.635,1.00) m/s in 50–59 year-old males to 0.656 (0.488,0.816) m/s in the 80+ group for the males and from 0.696 (0.548,0.909) m/s in 50–59 year-old females to 0.500 (0.3987,0.690) m/s in the 80+ group. These values were generally lower when compared to median gait speed values of 1.38 m/s, 1.30 m/s, 1.22 m/s and 1.08 m/s for men aged 60–65 years, 66–70 years, 71–75 years and ≥76 years respectively and 1.41 m/s, 1.27 m/s, 1.08 m/s and 0.92 m/s for women aged 60–65 years, 66–70 years, 71–75 years and ≥76 years respectively in a cross-sectional study conducted in Croatia [31]. The relatively lower gait speed probably has a strong correlation with the lower life expectancy of 64 and 65 years for Ghana and South Africa respectively whereas that of The Croatia stands at 76 years [5]. This is important especially as gait speed has important association with survival outcomes. South Africans had a significantly higher odds of having higher gait speeds compared to Ghana. This could be attributed to factors including the relatively higher health expenditure compared to Ghana. For example, in the years 2015 and 2020, South Africa recorded a health expenditure of US$ 499 and US$ 490 compared to Ghana’s US$ 78 and US$ 85 [32]. Investment in health care is associated with improved quality of life (QoL) and higher gait speed values are measurable outcomes that are related to QoL.

Age is an important non-modifiable predictor of gait speed. Increasing age has been found to be associated with decreasing gait speed across both males and females [14,16]. In this study, we observed that gait speed decreased from a mean of 0.84 to 0.55m/s as the individual advanced in age from 50 years. The reduction in gait speed was more pronounced among individuals above 80 years. This results is consistent with that recorded in The Netherlands where gait speed was found to decrease prominently especially from the age of 80 years and above compared to those in 60 years and above [33]. The effect was more prominent in females than in men. A closer look at the strong determinants of mortality in this sub-region is a crucial need to better guide the care of these older adults.

Gender is considered an important predictor of gait speed. Studies have shown that there is a significant difference in the gait between males and females with a slower gait speed observed in females than in males [33,34]. Averagely, maximum gait speed is about 19.2% lower in females than in males [35]. Lower physical exercise and higher BMI observed among older female adults aged 55 years and above compared to their male counterparts have been proposed as the possible explanation for the difference in gait between males and females [34]. In our study, we found the median gait speed for males to be higher than that of the females across all age groups by a significant factor of 0.55. Another important factor associated with gait speed is the residence of the older adult. The rural or urban settlement is considered a significant predictor of gait speed in older black older persons. The overall average walking speed of adult Vietnam community dwellers was 0.83 ± 0.27 m/s [29]. Gait speed has been observed to be higher among rural dwellers than urban dwellers [36,37]. However Aziz et al., found no significant difference in gait speed between rural dwellers and urban dwellers in the United States [38]. The gait speed of older rural dwellers in this study was higher than the gait speed of older urban dwellers. A plausible explanation for this observation may be based on the grounds that older rural dwellers in Sub-Saharan Africa are involved in a lot of farming and other physical activities keeping them strong and healthy than their counterparts in the urban areas.

For markers of anthropometric and functional status, BMI and WHODAS negatively correlated with gait speed in the Sub-Saharan African black population of the SAGE study as noted in other studies [16,39]. These factors are known predictors of impaired functional status and disability. Body Mass Index (BMI) is a modifiable risk factor for gait speed. The relationship between BMI and gait speed has been studied in different populations. Among the Latin Americans and the Caribbeans, M.A. Perez-Sousa et al. showed that sarcopenia and high BMI are associated with slower gait speed [40]. A study by Xin Fang et al., showed a slowed gait speed among obese and overweight Chinese participants [41]. In England, dynapenic abdominal obesity was associated with slower gait speed, which did not occur with dynapenic obesity measured by BMI [42]. The AMI study described a U-shaped relationship between gait speed and BMI among older adults residing in rural France [43]. The AMI study demonstrated that obese, overweight and underweight people have slow gait speed compared to individuals with normal BMI. Our study also describes a non-linear or U-shaped relationship between BMI and gait speed. We found a normal gait speed across all ages among individuals with normal BMI. However, Obesity and underweight older adults in Sub-Saharan Africa are associated with slower gait speed.

Self-reported disability and observed functional limitations are high among older adults in China, India, Russia, South Africa, Ghana, and Mexico [44]. Advanced age is associated with functional disability and higher WHODAS scores. In India, the mean WHODAS score was 34.2 for those aged 70–75 years but as high as 39.8 for those aged 75–80 years whereas in the Russian Federation the mean score was 27.0 for those aged 70–75 years, and 34.0 for those 75–80 years and in Ghana the mean score of 28.2 for those 70–75 years and 33.0 for those 75–80 years old [45]. However, a significantly lower level of disability was found in China with a mean score of 12.4 for those aged 70–75 years and 18.0 for those 75–80 years old [45]. In our study WHODAS scores are strongly associated with gait speed in the Sub-Saharan Africa and respondents in a higher WHODAS score category have lower odds of being in a higher gait speed category. Respondents in lower WHODAS score categories had higher gait speeds across the age groups compared to those with higher WHODAS scores. Cardiovascular risk factors including having a history of angina was found to be significantly associated with reduced gait speed in older adults. This finding is similar to that reported in Brazil where the authors found that factors including the presence of cardiovascular disease (OR = 2.15) were associated with lower gait speed in older adults aged 60 years and over in Brazil [16]. Gait speed is considered an important measure of the functional status of older adults and thus a vital sign in assessing older patients. We have provided specific normative data and for gait speed aimed at helping health professionals in screening functional dependence and monitoring the effectiveness of rehabilitative interventions among older patients. Normal walking speed should be further integrated into our clinics as part of our normal physical screenings and assessments of the health status of our older adults.

Also, racial disparity in gait speed has been observed among whites and blacks. Gait speed is observed to be about 0.08m/sec slower among blacks than Caucasians [39]. Blacks have a slower gait speed and they are more unlikely to complete their walking task than non-Hispanic Whites [46,47]. However, Taylor et al., have shown that racial disparity does not exist after controlling variables such as pain that are associated with gait speed [48]. This study demonstrates a significant difference observed between the gait speed among blacks and whites in Sub-Saharan Africa (Supporting information). Participants of the “white” race in South Africa recorded increased odds of having higher gait speed, by a factor of 2.68 (95%CI 2.09, 3.59, p<0.001), as opposed to African/Black. In contrast to the significant difference between the Caucasian and black cohorts in South Africa, we observed similar gait speeds among the Ghanaian Cohort and the South African Black cohort. This may explain the similarities in life expectancy in both countries which is about 64 and 65 years for Ghana and South Africa respectively [5].

## Conclusion

Gait speed in Sub-Saharan Africa is influenced by both modifiable and non-modifiable factors such as age, sex, race, BMI, rural or urban dwelling, WHODAS score, obesity, and the presence of cardiovascular disease. Advanced age, female sex, black race, obesity, urban settlement, higher WHODAS score, and the presence of cardiovascular disease risk factor (hypertension and diabetes) are associated with slower gait speed. Older adults especially urban dwellers are advised against a sedentary lifestyle. The normative data provide an assessment basis for programs aimed at reducing morbidity and mortality in the older adults. The functional status of the older adults can be assessed using their gait speed and therefore it is important to incorporate gait speed into our daily clinic assessment of vital signs most especially among older adults.

## Supporting information

S1 Checklist (DOCX)

S1 Table Association between selected predictors and gait speed in South Africa. ^a^- Classification adopted from [18]; OR = Odds Ratio; CI = Confidence Interval; *1* = Reference; p-value <0.05 was deemed significant.
(PDF)

S2 Table Interaction between ethnicity and age vs gait speed in the South African cohort. OR = Odds Ratio, CI = Confidence Interval and * = shows Interaction.
(PDF)

S3 Table Test for collinearity amongst predictors via variance inflation factor. (PDF)

S4 Table Test for proportional odds assumption: Demographic variables. *1* = Reference. Binary Logistic Regression Model 1 has two outcome levels: Gait speeds below the 25th percentile versus Gait Speeds above the 25th percentile. Binary Logistic Regression Model 2 has two outcome levels: Gait speeds below the 75th percentile versus Gait Speeds above the 75th percentile.
(PDF)

S5 Table Test for proportional odds assumption: WHODAS and BMI. *1* = Reference. Binary Logistic Regression Model 1 has two outcome levels: Gait speeds below the 25th percentile versus Gait Speeds above the 25th percentile. Binary Logistic Regression Model 2 has two outcome levels: Gait speeds below the 75^th^ percentile versus Gait Speeds above the 75^th^ percentile.
(PDF)
