# Supplementary material for: Gait speed and its associated factors among older black adults in Sub-Saharan Africa: Evidence from the WHO study on Global AGEing in older adults (SAGE)
Source: PLoS One. 2024 Apr 18;19(4):e0295520. doi: 10.1371/journal.pone.0295520 (PMC11025960; doi:10.1371/journal.pone.0295520)
Supplement: S1 Table — a- Classification adopted from [18]; OR = Odds Ratio; CI = Confidence Interval; 1 = Reference; p-value <0.05 was deemed significant. (PDF) [file pone.0295520.s002.pdf]

S1 Table

	Unadjusted Model			Adjusted Model	
**Characteristic**	**Median normal gait speed (m/s)**	**OR (95% CI)**	**p-value**	**OR (95% CI)**	**p-value**
**Ethnicity[a]**					
African/Black	0.667	*1*			
Coloured	0.714	1·35 (1·12,1·62)	0.001	1·52 (1·25,1·84)	<0·001
Indian/Asian	0.741	0·99 (0·77,1·26)	0.910	0·98 (0·76,1·26)	0·870
White	1.000	2.74 (2·09,3·59)	<0.001	3·06 (2·32,4·03)	<0·001
**Age**		0.97(0.96,0.97)	<0.001	0·97 (0·96,0·98)	<0·001
**Height**		1.06 (0.95,1.19)	0.266		
**BMI**					
Normal Weight	0.784	*1*		*1*	
Obese	0.667	0.52 (0·43,0·62)	<0.001	0·51 (0·42,0·61)	<0·001
Overweight	0.769	0.99 (0·82,1·21)	0.945	0·96 (0·79, 1·18)	0·720
Underweight	0.755	0.77 (0·57,1·04)	0.085	0·82 (0·60, 1·11)	0·200
**Income (in quintiles)**					
1st	0.690	*1*			
2nd	0.741	1·08(0.86,1.36)	0.486		
3rd	0.667	0·91 (0.72,1.13)	0.391		
4th	0.667	0·72 (0.57,0.90)	<0.005		
5th	0.762	0·96 (0.76,1.20)	0.714		
**History of Hypertension**					
Yes	0.667	*1*		*1*	
No	0.741	1.54(1.32,1.80)	<0.001	1·27 (1·08, 1·50)	<0·005
**History of Diabetes Mellitus**					
Yes	0.667	*1*		*1*	
No	0.702	1.70(1.34,2.15)	<0.001	1·38 (1·07, 1·78)	0·013
**History of Angina**					
Yes	0.667	*1*		*1*	
No	0.702	1.30(1.09,1.55)	<0.005	1·36 (1·12, 1·65)	< 0.005
