# Supplementary material for: Gait speed and its associated factors among older black adults in Sub-Saharan Africa: Evidence from the WHO study on Global AGEing in older adults (SAGE)
Source: PLoS One. 2024 Apr 18;19(4):e0295520. doi: 10.1371/journal.pone.0295520 (PMC11025960; doi:10.1371/journal.pone.0295520)
Supplement: S2 Table — OR = Odds Ratio, CI = Confidence Interval and * = shows Interaction. (PDF) [file pone.0295520.s003.pdf]

S2 Table

Characteristic	OR(95% CI)	p-value
Ethnicity * Age		
Coloured * Age	0·98 (0·96,0·99)	p=0.0136
Indian/Asian * Age	1·00 (0·97,1·03)	p=0.939
White * Age	0·96 (0·93,0·99)	p=0.006
