# Supplementary material for: Gait speed and its associated factors among older black adults in Sub-Saharan Africa: Evidence from the WHO study on Global AGEing in older adults (SAGE)
Source: PLoS One. 2024 Apr 18;19(4):e0295520. doi: 10.1371/journal.pone.0295520 (PMC11025960; doi:10.1371/journal.pone.0295520)
Supplement: S3 Table — (PDF) [file pone.0295520.s004.pdf]

S3 Table

**Predictor variable**	**Variance inflation factor**	**Degrees of freedom**	**Generalised variance inflation factor**
Age	1.037400	1	1.018529
Residence	1.081021	1	1.039722
Sex	1.042416	1	1.020988
Height	1.034678	1	1.017191
