# Supplementary material for: Gait speed and its associated factors among older black adults in Sub-Saharan Africa: Evidence from the WHO study on Global AGEing in older adults (SAGE)
Source: PLoS One. 2024 Apr 18;19(4):e0295520. doi: 10.1371/journal.pone.0295520 (PMC11025960; doi:10.1371/journal.pone.0295520)
Supplement: S4 Table — 1 = Reference. Binary Logistic Regression Model 1 has two outcome levels: Gait speeds below the 25th percentile versus Gait Speeds above the 25th percentile. Binary Logistic Regression Model 2 has two outcome levels: Gait speeds below the 75th percentile versus Gait Speeds above the 75th percentile. (PDF) [file pone.0295520.s005.pdf]

S4 Table

	**Odds Ratios: Ordinal Logistic Regression Model**	**Odds Ratios: Binary Logistic Regression Model 1**	**Odds Ratios: Binary Logistic Regression Model 2**
**Age**	0.9620149	0.9585446	0.9666483
**Sex**			
Male		*1*	
Female	0.5558908	0.5252033	0.5851526
**Residence**			
Urban		*1*	
Rural	1.4413524	1.5450683	1.3175685
