# Supplementary material for: Gait speed and its associated factors among older black adults in Sub-Saharan Africa: Evidence from the WHO study on Global AGEing in older adults (SAGE)
Source: PLoS One. 2024 Apr 18;19(4):e0295520. doi: 10.1371/journal.pone.0295520 (PMC11025960; doi:10.1371/journal.pone.0295520)
Supplement: S5 Table — 1 = Reference. Binary Logistic Regression Model 1 has two outcome levels: Gait speeds below the 25th percentile versus Gait Speeds above the 25th percentile. Binary Logistic Regression Model 2 has two outcome levels: Gait speeds below the 75th percentile versus Gait Speeds above the 75th percentile. (PDF) [file pone.0295520.s006.pdf]

S5 Table

	**Odds Ratios: Ordinal Logistic Regression Model**	**Odds Ratios: Binary Logistic Regression Model 1**	**Odds Ratios: Binary Logistic Regression Model 2**
**WHODAS score**			
None/Mild		*1*	
Moderate	0.3883620	0.3203590	0.5007623
Severe/Extreme	0.2108076	0.1685264	0.3779465
**BMI**			
Normal weight		*1*	
Underweight	0.8616382	0.8624994	0.8542009
Overweight	0.9523330	0.8710294	1.0250234
Obese	0.5206624	0.4067082	0.7014013
